# CSF1/CSF1R signaling mediates malignant pleural effusion formation

**DOI:** 10.1172/jci.insight.155300

**Published:** 2022-03-22

**Authors:** Chrysavgi N. Kosti, Photene C. Vaitsi, Apostolos G. Pappas, Marianthi P. Iliopoulou, Katherina K. Psarra, Sophia F. Magkouta, Ioannis T. Kalomenidis

**Affiliations:** 1 Marianthi Simou Laboratory, 1st Department of Critical Care and Pulmonary Medicine, School of Medicine, National and Kapodistrian University of Athens, Athens, Greece.; 2 Department of Immunology — Histocompatibility, Evangelismos Hospital, Athens, Greece.

**Keywords:** Angiogenesis, Oncology, Cancer, Macrophages

## Abstract

Malignant pleural effusion (MPE) is an incurable common manifestation of many malignancies. Its formation is orchestrated by complex interactions among tumor cells, inflammatory cells, and the vasculature. Tumor-associated macrophages present the dominant inflammatory population of MPE, and M2 macrophage numbers account for dismal prognosis. M2 polarization is known to be triggered by CSF1/CSF1 receptor (CSF1R) signaling. We hypothesized that CSF1R^+^ M2 macrophages favor MPE formation and could be therapeutically targeted to limit MPE. We generated mice with CSF1R-deficient macrophages and induced lung and colon adenocarcinoma–associated MPE. We also examined the therapeutic potential of a clinically relevant CSF1R inhibitor (BLZ945) in lung and colon adenocarcinoma–induced experimental MPE. We showed that CSF1R^+^ macrophages promoted pleural fluid accumulation by enhancing vascular permeability, destabilizing tumor vessels, and favoring immune suppression. We also showed that CSF1R inhibition limited MPE in vivo by reducing vascular permeability and neoangiogenesis and impeding tumor progression. This was because apart from macrophages, CSF1R signals in cancer-associated fibroblasts leading to macrophage inflammatory protein 2 secretion triggered the manifestation of suppressive and angiogenic properties in macrophages upon CXCR2 paracrine activation. Pharmacological targeting of the CSF1/CSF1R axis can therefore be a vital strategy for limiting MPE.

## Introduction

Malignant pleural effusion (MPE) is a common complication of intra- and extrathoracic malignancies. It is associated with advanced stage, poor survival, and compromised quality of life (1). Intrapleural tunneled catheters and pleurodesis present the standard of care for MPE (2), and they offer symptom relief in most cases. However, they are invasive, not always effective, and not free of complications (2). There is thus an urgent need for a shift to a curative approach targeting fluid formation. In this direction, unraveling the pathogenetic mechanisms of MPE becomes a key area of focus.

MPE formation is regulated by interactions among tumor cells, the host vasculature, and immune system, causing vascular hyperpermeability, angiogenesis, and inflammatory responses (3). Tumor-associated macrophages (TAMs) present the dominant cell population of MPE (4–6) and fluid M2 macrophage counts have recently been associated with impaired survival in patients with MPE (5, 7). M2 macrophages promote all processes favoring MPE formation. They stimulate angiogenesis by secreting several angiogenic factors and proteases (8, 9) and orchestrate immune suppression through hampering effective T cell responses (6, 10, 11). The aforementioned observations suggest that repolarization of the pleural macrophages might limit MPE.

Polarization of TAMs toward the M2 phenotype is driven by the CSF1 (mainly secreted by tumor cells)/CSF1 receptor (CSF1R) axis (12, 13). We thus hypothesized that a) CSF1R^+^ M2 macrophages enhance experimental MPE formation, mainly by modulating the immune tumor/pleural microenvironment to promote angiogenesis and vessel permeability and b) that CSF1R inhibition would effectively limit MPE formation.

## Results

### CSF1R^+^ macrophages promote MPE by enhancing pleural vascular permeability and destabilizing tumor endothelium.

In order to uncover the role of CSF1R-expressing macrophages in MPE formation, we created *CSF1R^fl/fl^* *LysM-Cre^hemi^* transgenic mice in which CSF1R expression had been specifically ablated in monocytes/macrophages (referred to as CSF1R^–^ macrophages hereafter) and respective control *CSF1R^fl/fl^ LysM-Cre^–/–^* (referred to as CSF1R^+^ macrophages hereafter). We subsequently used 2 murine adenocarcinoma cell lines to create MPE. As expected, CSF1R expression was downregulated in pleural and tumor macrophages of *CSF1R^fl/fl^* *LysM-Cre^hemi^* mice (Supplemental Figure 1, A and B; supplemental material available online with this article; https://doi.org/10.1172/jci.insight.155300DS1). CSF1R was expressed by 50%–72% of TAMs, fewer than 5% of LY6C^+^ monocytes, and 6%–25% of DCs (Supplemental Figure 2, A–C). CSF1R deletion was most profound in macrophages (Supplemental Figure 2, A–E). CSF1R deletion in monocytes and DCs was insignificant (Supplemental Figure 2, B and C). Granulocytes and lymphocytes did not express the receptor (Supplemental Figure 2, D and E). Overall, use of *CSF1R^fl/fl^* *LysM-Cre^hemi^* mice allowed us to selectively delete CSF1R in macrophages.

**Figure 1:**
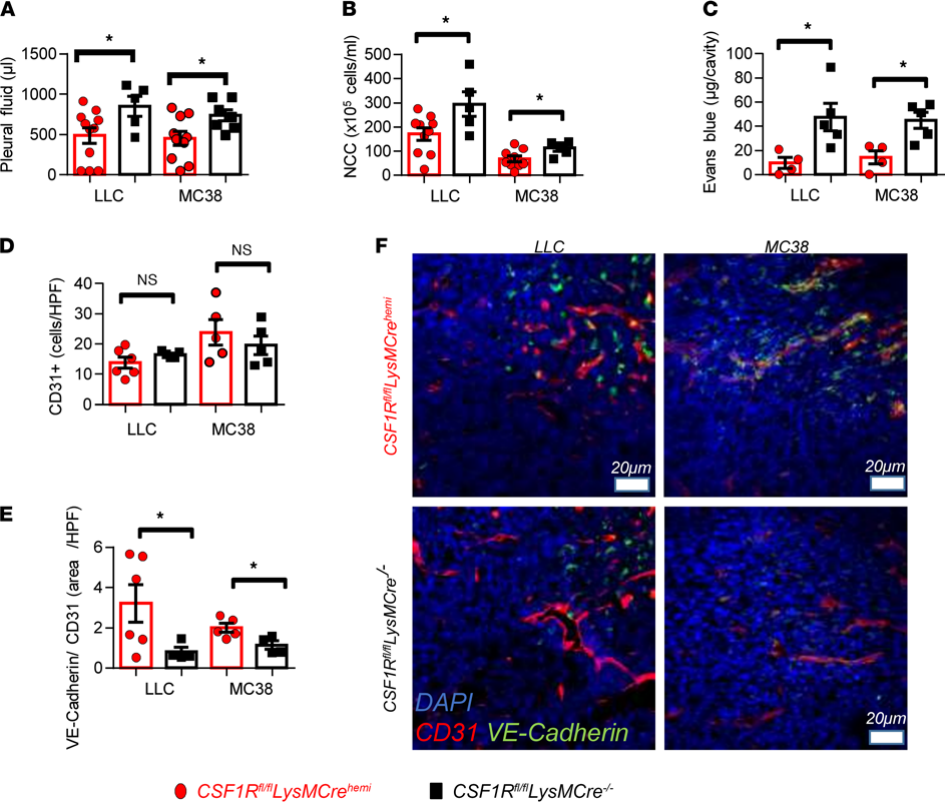
CSF1R^+^ macrophages promote MPE accumulation by enhancing pleural vascular permeability and destabilizing tumor vessels. C57BL/6 mice whose macrophages were devoid of CSF1R (*LysM-Cre^hemi^* *CSF1R^fl/fl^*) were created as described in Methods. *LysM-Cre^–/–^* *CSF1R^fl/fl^* mice, whose macrophages are able to express CSF1R, were used as positive controls. MPE was induced upon intrapleural delivery of 1.5 × 10^5^ Lewis lung cells (LLCs) or MC38 murine adenocarcinoma cells. Animals were euthanized 13 days later. (**A**) Pleural fluid was retrieved and quantified. (**B**) Total pleural cell numbers were determined under a hematocytometer. Data presented as mean ± SEM. LLC: *LysM-Cre^hemi^* *CSF1R^fl/fl^* *n* = 11, *LysM-Cre^–/–^* *CSF1R^fl/fl^* *n* = 5. MC38: *LysM-Cre^hemi^* *CSF1R^fl/fl^* *n* = 10, *LysM-Cre^–/–^* *CSF1R^fl/fl^* *n* = 7. *P < 0.05 compared with *LysM-Cre^hemi^* *CSF1R^fl/fl^* by 2-tailed Student’s *t* test. (**C**) Pleural vascular permeability was evaluated after intrapleural injection of Evans blue. Data presented as mean ± SEM. LLC: *LysM-Cre^hemi^* *CSF1R^fl/fl^* *n* = 4, *LysM-Cre^–/–^* *CSF1R^fl/fl^* *n* = 5. MC38: *LysM-Cre^hemi^* *CSF1R^fl/fl^* *n* = 4, *LysM-Cre^–/–^* *CSF1R^fl/fl^* *n* = 5. *P < 0.05 compared with *LysM-Cre^hemi^* *CSF1R^fl/fl^* by 2-tailed Student’s *t* test. (**D** and **F**) Tumor vessel networks were visualized upon CD31 immunofluorescence staining. (**E** and **F**) Vascular normalization was assessed by the ratio of VE-cadherin^+^CD31^+^ tumor areas quantified using the ImageJ software (NIH). (**F**) Representative images of VE-cadherin expression (green) by tumor vessels (red). Scale bar: 20 μm. Data presented as mean ± SEM. LLC: *LysM-Cre^hemi^* *CSF1R^fl/fl^* *n* = 6, *LysM-Cre^–/–^* *CSF1R^fl/fl^* *n* = 4. MC38: *LysM-Cre^hemi^* *CSF1R^fl/fl^* *n* = 5, *LysM-Cre^–/–^* *CSF1R^fl/fl^* *n* = 5. *P < 0.05 compared with *LysM-Cre^hemi^* *CSF1R^fl/fl^* by 2-tailed Student’s *t* test.

Mice with CSF1R^+^ macrophages presented significantly larger MPEs (Figure 1A), more pleural fluid nucleated cells (Figure 1B), and enhanced pleural vascular permeability (Figure 1C) compared with the control animals. Notably, enumeration of tumor foci on the lungs suggested that intrapleural tumor dissemination was not altered (Supplemental Figure 1C). To reveal possible changes of the microvasculature, tumor vessel networks were visualized upon CD31 staining, and vascular integrity was evaluated using the endothelial specific junctional marker VE-cadherin. No difference in vessel density was found (Figure 1, D and F), but CSF1R^+^ macrophages were found to destabilize tumor vessels, as demonstrated by the significant reduction of vascular VE-cadherin (Figure 1, E and F).

We then explored whether macrophage CSF1R silencing affects inflammatory MPE-related aspects. Pleural macrophage concentrations did not differ between groups (Supplemental Figure 3, A and B). Nevertheless, mice with CSF1R-deficient macrophages demonstrated a shift toward the M1 phenotype of pleural but not tumor macrophages (Supplemental Figure 3, C and D). In addition, absence of CSF1R^+^ expression by macrophages was associated with reduced myeloid-derived suppressor cells (MDSCs; Supplemental Figure 4, A and B), increased tumor CD8^+^ cell infiltration, and enhanced CD8^+^ cell activation in tumors (Supplemental Figure 4, C–E). Pleural and tumor levels of MPE-related proinflammatory mediators (IL-6; VEGF; monocyte chemoattractant protein-1, MCP-1; TNFA; secreted phosphoprotein 1, OPN; and macrophage inflammatory protein 2, MIP2) were not affected by macrophage CSF1R ablation (Supplemental Figure 5, A–D).

Collectively, these results suggest that CSF1R^+^ macrophages promote MPE mainly by inducing vascular hyperpermeability, destabilizing tumor vessels, and favoring immune suppression. In addition, they provide a rationale for pharmacological targeting of CSF1R for MPE treatment.

### CSF1R inhibitor administration limits MPE and pleural tumor dissemination by attenuating tumor angiogenesis and enhancing tumor cell apoptosis in vivo.

**Figure 2:**
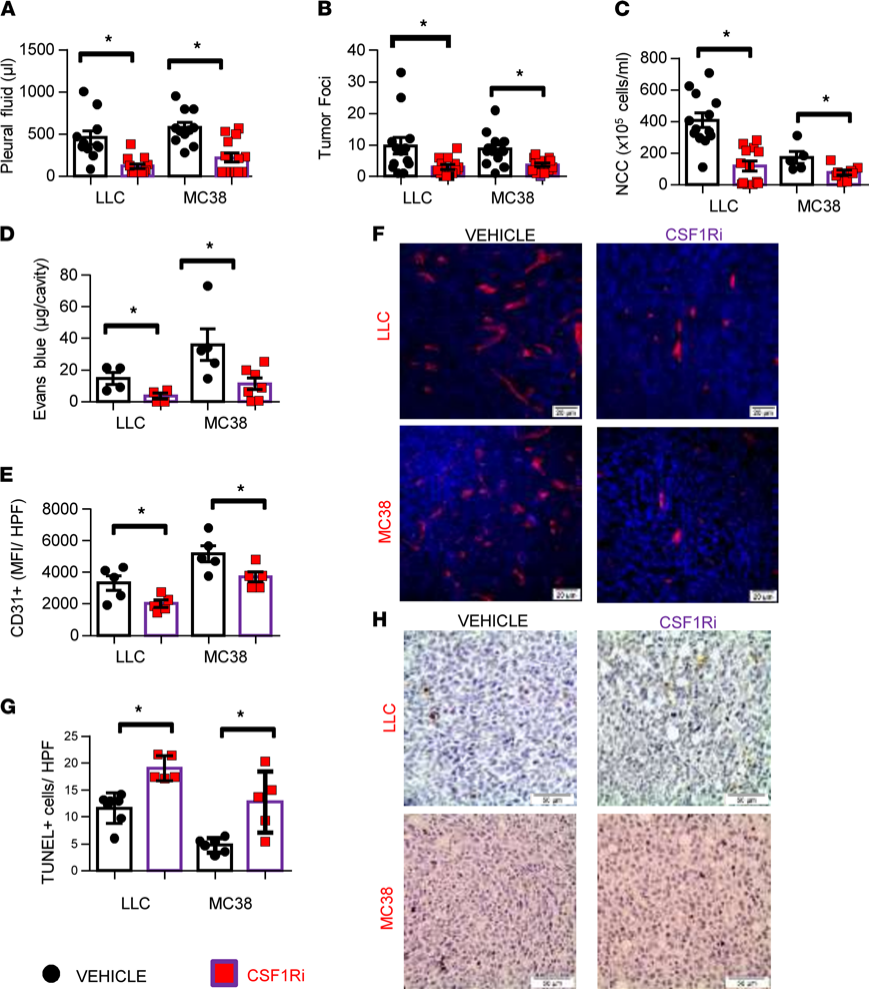
Pharmacological targeting of CSF1R abrogates MPE formation. C57BL/6 mice bearing LLC- or MC38- induced MPE were po administered the CSF1R inhibitor BLZ945 (200 mg/kg body weight, once daily) or vehicle starting 4 days upon initiation of the experiment. Animals were euthanized 13 days later. (**A**) Pleural fluid was retrieved, and its volume was measured. (**B**) Lungs were harvested and lung surface tumor foci were counted under a stereoscope. Data presented as mean ± SEM. LLC: vehicle *n* = 13, CSF1Ri *n* = 13. MC38: vehicle *n* = 13, CSF1Ri *n* = 14. *P < 0.05 compared with vehicle by 2-tailed Student’s *t* test. (**C**) Total pleural cell numbers were determined under a hematocytometer. Data presented as mean ± SEM. LLC: vehicle *n* = 13, CSF1Ri *n* = 13. MC38: vehicle *n* = 5, CSF1Ri *n* = 8. *P < 0.05 compared with vehicle by 2-tailed Student’s *t* test. NCC, nucleated cell counts. (**D**) Pleural vascular permeability was evaluated using the Evans blue method. Data presented as mean ± SEM. LLC: vehicle *n* = 4, CSF1Ri *n* = 4. MC38: vehicle *n* = 5, CSF1Ri *n* = 7. *P < 0.05 compared with vehicle by 2-tailed Student’s *t* test. (**E** and **F**) Tumor tissue sections of vehicle and CSF1Ri-treated animals were stained for CD31 and vessel density was determined using ImageJ software. Data presented as mean ± SEM. *n* = 5 for all groups, *P < 0.05 compared with vehicle by 2-tailed Student’s *t* test. (**G** and **H**) Apoptotic tumor cells were evaluated in tissue sections by TUNEL assay. Data presented as mean ± SEM. LLC: vehicle *n* = 7, CSF1Ri *n* = 5. MC38: vehicle *n* = 6, CSF1Ri *n* = 5. *P < 0.05 compared with vehicle by 2-tailed Student’s *t* test. Scale bars: 20 μm (**F**), 50 μm (**H**). HPF, high-power field.

To examine how pharmacological disruption of CSF1R signaling affects MPE accumulation, we treated LLC-induced and MC38-induced MPE-bearing mice with BLZ945, a highly selective small-molecule inhibitor of CSF1R (hereafter referred to as CSF1Ri) or vehicle. CSF1R inhibition reduced MPE size (Figure 2A) and intrapleural tumor spread (Figure 2B and Supplemental Figure 6). Pleural fluid cell counts of CSF1Ri-treated mice were also decreased (Figure 2C). In addition, CSF1Ri inhibited pleural vascular hyperpermeability (Figure 2D) and tumor angiogenesis (Figure 2, E and F), both critical aspects of MPE pathogenesis (3). Although no significant alteration in tumor proliferation was observed (data not shown), CSF1Ri-treated tumors presented higher apoptosis rates (Figure 2, G and H). Notably, neither of the 2 tumor cell lines expressed the receptor (data not shown), and their viability was not affected by CSF1Ri in vitro (Supplemental Figure 7). These observations argue against an autocrine or a paracrine effect of the CSF1/CSF1R axis on tumor cells themselves, implying that tumor and MPE-limiting properties of CSF1Ri should be attributed to its impact on the tumor microenvironment.

### CSF1R inhibition attenuates pleural macrophage accumulation, diminishes their angiogenic potential, and stimulates their M1 polarization.

**Figure 3:**
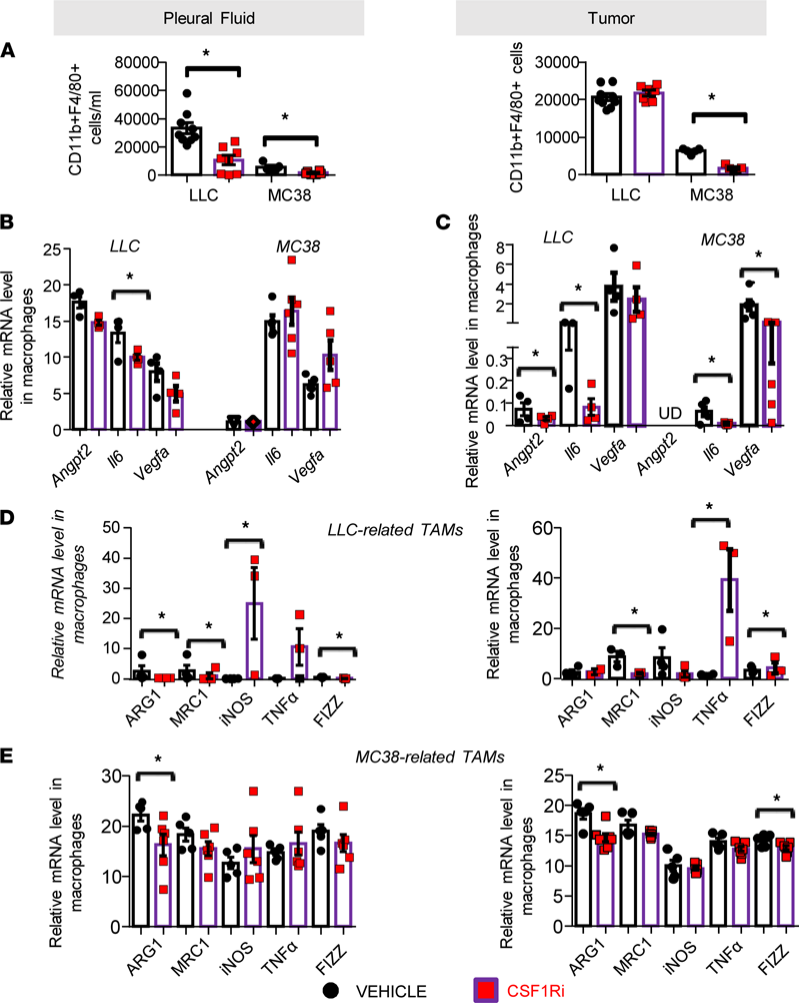
Pharmacological targeting of CSF1R reduced pleural TAMs and attenuated their M2 polarization and their secretion of proangiogenic mediators. (**A**) Pleural (left) and tumor (right) cells were stained for CD45, CD11b, and F4/80 and analyzed by flow cytometry. Data presented as mean ± SEM. LLC: vehicle *n* = 9, CSF1Ri *n* = 8. MC38: vehicle *n* = 6, CSF1Ri *n* = 6. *P < 0.05 compared with vehicle by 2-tailed Student’s *t* test. (**B**–**E**) TAMs were isolated using anti-F4/80–loaded magnetic beads, and mRNA levels of (**B** and **C**) angiogenic (angiopoietin 2, *Angpt2*), *Il6*, and *Vegfa* genes were determined by real-time PCR. Data presented as mean ± SEM. LLC: *n* = 4 for both groups. MC38: vehicle *n* = 5 for both groups. *P < 0.05 compared with vehicle by 2-tailed Student’s *t* test. (**D** and **E**) M2 (*Arg1*, *Mrc1*) and M1 (*Fizz*, *Tnfa*, *Inos*) genes were also quantified by real-time PCR. Data presented as mean ± SEM. LLC: vehicle *n* = 4–5, CSF1Ri *n* = 5. MC38: vehicle *n* = 4–5, CSF1Ri *n* = 6. *P < 0.05 compared with vehicle by 2-tailed Student’s *t* test. UD, undetectable.

CSF1R inhibition reduced pleural macrophage accumulation (Figure 3A). Tumor macrophage numbers were reduced only in the MC38 model (Figure 3B). Having shown that CSF1Ri inhibited vascular permeability and angiogenesis, we examined whether the inhibitor affected TAM expression of major regulators of aberrant angiogenesis: *Vegfa* (promoting leaky neovessel formation; ref. 14), *Il6*, and *Angpt2* (favoring sprouting and destabilization; refs. 14, 15). Tumor macrophages of CSF1Ri-administered animals exhibited significantly lower expression of *Il6* and *Vegfa* (in MC38 model) or *Il6* and *Angpt2* (in LLC model) (Figure 3C). *Il6* expression was reduced in pleural macrophages of LLC-induced MPE-bearing mice (Figure 3B). We also hypothesized that macrophages from CSF1Ri-treated mice might be functionally altered. To examine the effects of CSF1Ri treatment in TAM phenotypes, we determined a 5-gene signature of key functional macrophage markers in isolated tumor and pleural fluid TAMs. As shown in Figure 3D, in LLC-related pleural TAMs in vivo, CSF1R blockade promoted M1 *iNOS* and *Fizz* marker expression and downregulated the M2-associated *Arg1* and *Mrc1* genes. In respective tumor macrophages, *Tnfa* and *Fizz* M1 markers were upregulated and the Mrc1 M2 gene was downregulated. Similarly, pleural and tumor MC38-related TAMs were found to express less M2 *Arg1* gene upon CSF1R blockade (Figure 3E).

### CSF1R inhibition reshapes the pleural immune environment by limiting myeloid-suppressing populations, enhancing DC and CD8^+^ lymphocyte activation, and reducing MPE-promoting chemo/cytokines.

**Figure 4:**
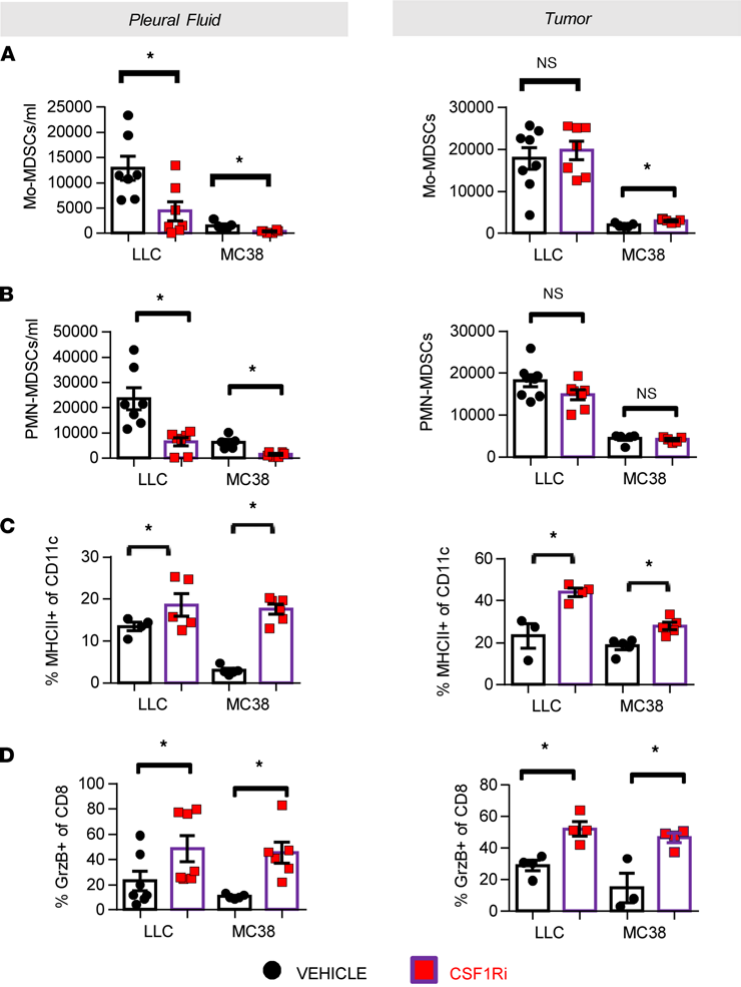
Pharmacological targeting of CSF1R reshapes the immune environment by limiting myeloid-suppressing populations and enhancing DC and CD8^+^ lymphocyte activation. Pleural (left) and tumor (right) abundance of myeloid-derived suppressor cells (MDSCs) of the monocytic (CD11b^+^Ly6C^+^) (**A**) and granulocytic (CD11b^+^Ly6G^+^) (**B**) lineage was determined in CSF1Ri- and vehicle-treated animals by flow cytometry. Data presented as mean ± SEM. LLC: vehicle *n* = 7, CSF1Ri *n* = 7. MC38: vehicle *n* = 5, CSF1Ri *n* = 5. *P < 0.05 compared with vehicle by 2-tailed Student’s *t* test. Mo, monocytic. (**C**) Activated (MHCII^+^) DCs (CD45^+^CD11C^+^) were also evaluated in pleural fluid and tumors of vehicle and CSF1Ri animals. Data presented as mean ± SEM. LLC: vehicle *n* = 4, CSF1Ri *n* = 5. MC38: vehicle *n* = 5, CSF1Ri *n* = 5. *P < 0.05 compared with vehicle by 2-tailed Student’s *t* test. (**D**) Activated (granzyme-B^+^) CD8^+^ lymphocytes were quantified as well in pleural fluid and tumors of vehicle and CSF1Ri animals. Data presented as mean ± SEM. LLC: vehicle *n* = 4–7, CSF1Ri *n* = 4–7. MC38: vehicle *n* = 3–5, CSF1Ri *n* = 4–6. *P < 0.05 compared with vehicle by 2-tailed Student’s *t* test.

We next explored the impact of CSF1Ri on additional myeloid immune populations potentially affected by CSF1R signaling. Pleural MDSCs of the monocytic (CD11b^+^Ly6C^+^, Figure 4A) and granulocytic (CD11b^+^Ly6G^+^, 4B) lineage were critically decreased upon CSF1Ri administration. Furthermore, CSF1R inhibition enhanced antigen presentation by pleural and tumor DCs, as indicated by higher MHCII expression (Figure 4C). Although lymphocyte infiltration was not increased after CSF1Ri therapy (data not shown), activation of pleural and tumor cytotoxic T cells was significantly enhanced (Figure 4D).

**Figure 5:**
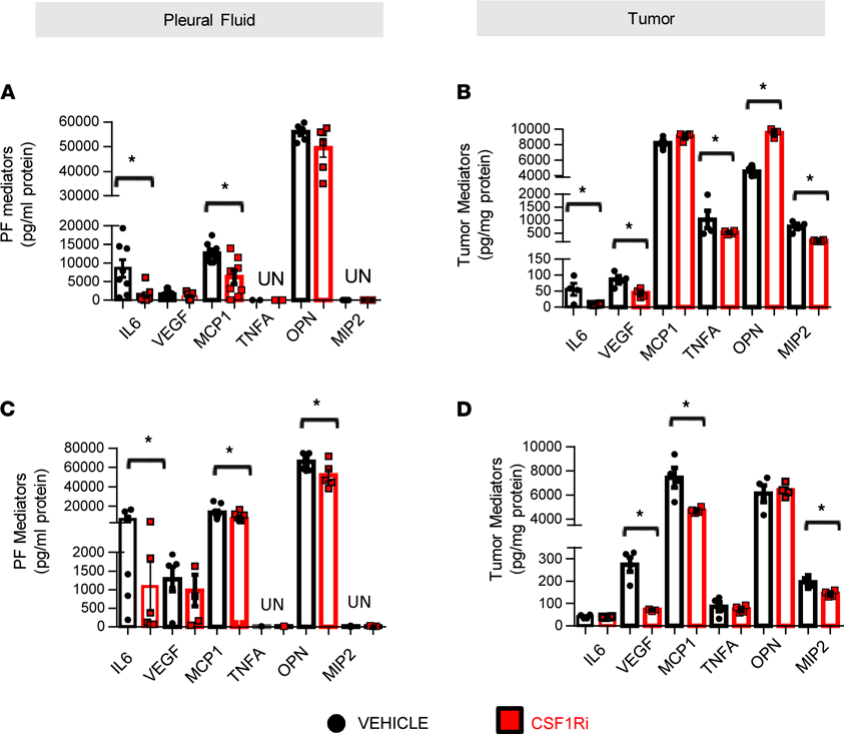
CSF1R inhibition reduces MPE-promoting pleural space chemokines. Key mediators proven to be associated with MPE progression IL-6, VEGF, MCP-1, TNFA, OPN, and MIP2 were quantified in the pleural fluid (**A** and **C**) and tumor lysates (**B** and **D**) of CSF1Ri- and vehicle-treated animals using ELISA. Data presented as mean ± SEM. LLC: vehicle *n* = 4–8, CSF1Ri *n* = 4–7. MC38: vehicle *n* = 5, CSF1Ri *n* = 5. *P < 0.05 compared with vehicle by 2-tailed Student’s *t* test.

Finally, since the cytokine/chemokine content contributes to the immunosuppressive milieu (16), we sought to quantify key mediators previously connected with MPE formation (3). CSF1Ri-treated tumors had significantly lower levels of VEGF and MIP2 (Figure 5, B and D). MCP-1 levels were significantly reduced only in MC38 tumors (Figure 5D) in accordance with the observed reduction of macrophage populations (Figure 3A). In LLC tumors, IL-6 and TNFA content were significantly decreased by CSF1Ri treatment. Levels of MCP-1 and IL-6 or OPN (Figure 5) were also decreased in the pleural fluid of CSF1Ri-treated groups.

### CSF1R inhibition impedes fibro-inflammatory stroma-associated macrophage suppression.

**Figure 6:**
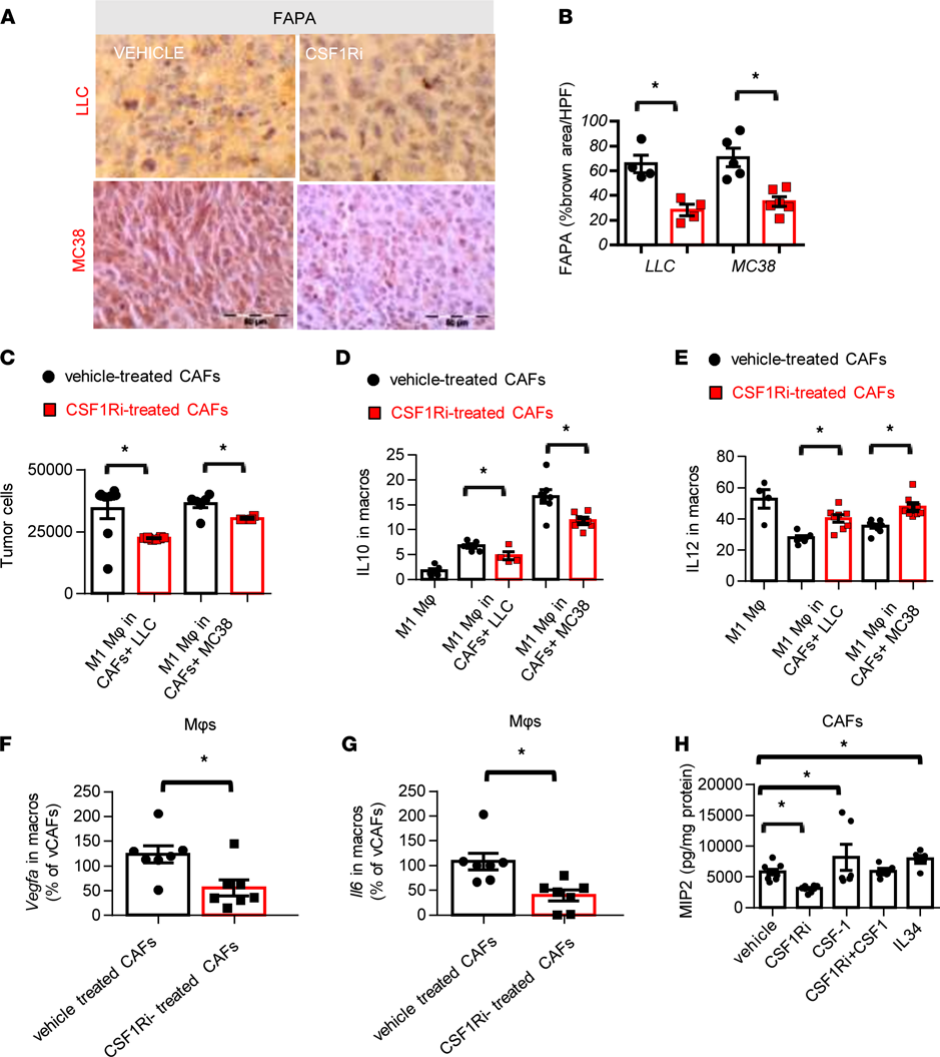
CSF1R inhibition reduces cancer-associated fibroblasts, which stimulates protumor effects of macrophages through the MIP2/CXCR2 axis. (**A** and **B**) CAFs in tumors of vehicle- and CSF1Ri-treated animals were visualized upon FAPA staining. (**A**) Representative pictures. Scale bar: 50 μm. (**B**) Results of image analysis. Data presented as mean ± SEM. LLC: *n* = 4 for both groups. MC38: *n* = 5 for both groups. *P < 0.05 compared with vehicle by 2-tailed Student’s *t* test. HPF, high-power field. (**C**) Activated CAFs isolated from LLC or MC38 tumors were treated with vehicle or 6700 nM CSF1Ri overnight and subsequently loaded with 7 × 10^4^ tumor cells/well; 8 hours later 2 × 10^5^ M1 macrophages/well were added to the tumor cell–CAF cocultures; 24 hours later, tumor cells were counted. (**D** and **E**) CD45^+^CD11b^+^F4/80^+^ macrophages were analyzed for IL-10 (**D**) and IL-12 (**E**) expression. Data presented as mean ± SEM, 2 independent experiments. LLC: vehicle-treated CAFs *n* = 5, CSF1Ri-treated CAFs *n* = 5. MC38: vehicle-treated CAFs *n* = 7, CSF1Ri-treated CAFs *n* = 7. *P < 0.05 compared with vehicle-treated CAFs by 2-tailed Student’s *t* test. (**F** and **G**) Isolated CAFs from LLC and MC38 tumors (treated with vehicle or CSF1Ri overnight) were cocultured with macrophages for 48 hours. Macrophages were isolated and mRNA levels of *Vegfa* (**F**) and *Il6* (**G**) were quantified by real-time PCR. Data presented as mean ± SEM. *n* = 7 for both groups, *P < 0.05 compared with indicated group by 2-tailed Student’s *t* test. (**H**) Isolated CAFs from LLC or MC38 tumors were treated with vehicle, CSF1Ri, CSF1, or IL-34 or pretreated with CSF1Ri for 2 hours and then CSF1 was added; 24 hours later, MIP2 levels were quantified in supernatants. Results were normalized to total protein. Data presented as mean ± SEM. Vehicle *n* = 6, CSF1Ri *n* = 6, CSF1 *n* = 5, CSF1Ri+CSF1 *n* = 4, IL-34 *n* = 5 (3 independent experiments). *P < 0.05 compared with indicated group by 1-way ANOVA (with Tukey’s post hoc test).

As shown above, the genetic ablation of CSF1R^+^macrophages and the CSF1Ri treatment limited MPE, while tumor pleural dissemination was reduced only by the inhibitor. This led us to assume that apart from macrophages, other CSF1R-expressing cells might be affected by the treatment. CSF1R is expressed by cancer-associated fibroblasts (CAFs) and mediates their tumor-promoting phenotype (17). We therefore sought to quantify activated tumor CAFs upon fibroblast activation protein-α (FAPA) staining (18). As presented in Figure 6, A and B, CSF1R blockade reduced CAFs in both adenocarcinoma models, while tumor CAF infiltration was not affected in the genetic CSF1R-ablated models (Supplemental Figure 8). CSF1Ri did not affect CAF viability in vitro (data not shown). We therefore focused on tumor-CAF-macrophage interplay and hypothesized that blockade of CSF1R/CSF1 signaling at CAFs might reverse their protective role on tumor cells. Heterotypic interaction among CAFs and macrophages was corroborated in triple cocultures (CAFs, tumor cells, and macrophages). M1 macrophage cytotoxicity against tumor cells was more efficient when CSF1R was inactivated in CAFs (Figure 6C). Moreover, CSF1R inhibition in CAFs conferred a significant reduction of IL-10 and an increase in IL-12 production in macrophages (Figure 6, D and E). Interestingly, CAFs were also found to affect the angiogenic potential of macrophages since *Vegfa* and *Il6* expression was reduced in macrophages that were cocultured with CSF1Ri-treated CAFs (Figure 6, F and G). Recent attention drawn to CAF-macrophage interactions has unveiled the importance of IL-6 and IL-8 as messengers between these 2 types (19). We therefore examined a panel of 6 mediators (VEGF, MIP2, IL-6, SDF1, TNFA, OPN) known to be secreted by CAFs to find those that are mediated by CSF1R activation. In our study, MIP2 secretion by CAFs was the only one found to be both enhanced upon CSF1R activation by CSF1 or IL-34 and repressed upon CSF1R blockade (Figure 6H) and could therefore be implicated in the heterotypic interactions described in Figure 6, C–G.

### CSF1R-associated CAF-suppressive effects are mediated by MIP2/CXCR2 signaling in macrophages.

**Figure 7:**
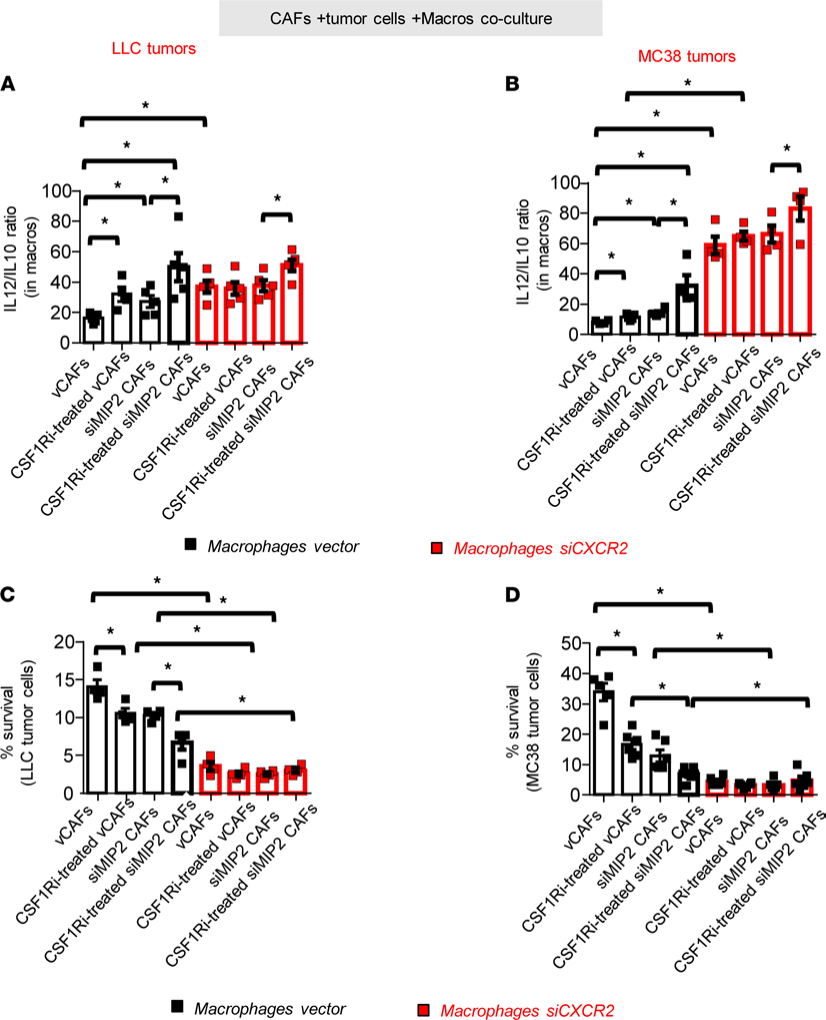
Suppressive effects of CAFs on macrophages are mediated through CAF-derived MIP2 expression mediated through macrophage MIP2 receptor. (**A** and **B**) Isolated CAFs with inherent (vCAFs) or silenced MIP2 expression (shMIP2 CAFs) and bone marrow–derived macrophages with inherent (macrophage vector) or silenced MIP2 receptor (macrophage shCXCR2) were used to repeat the setting described in Figure 6C. (**A** and **B**) CD45^+^CD11b^+^F4/80^+^macrophages were analyzed for IL-12/IL-10 ratio expression using flow cytometry and (**C** and **D**) tumor cells were counted using BCECF-AM measurement. Data presented as mean ± SEM, *n* = 4 for all groups (2 independent experiments). *P < 0.05 compared with indicated group by 1-way ANOVA (with Tukey’s post hoc test).

**Figure 8:**
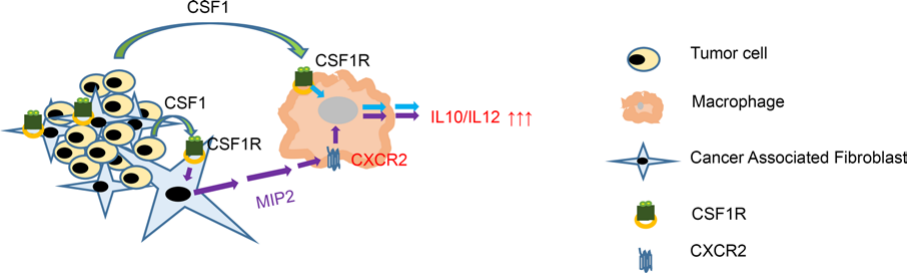
Schematic representation of intracellular and heterotypic intercellular CSF1R signaling. In macrophages, CSF1R activation by tumor cell–derived CSF1 triggers increase of the IL-10/IL-12 ratio, functionally associated with M2 responses. Activation of CSF1R residing on CAFs triggers MIP2 secretion by them. MIP2 signals through CXCR2 residing on macrophages, leading to IL-10/IL-12 ratio increase.

Having shown that MIP2 secretion by CAFs was stimulated by CSF1R activation, we tested whether disruption of this CAF-macrophage crosstalk could restore cytotoxicity of the latter. To elucidate this, we generated MIP2-deficient CAFs and CXCR2- deficient (MIP2 receptor–deficient) macrophages and repeated experiments. As expected, blockade of either CSF1R (in CAFs), or MIP2 (in CAFs), or CXCR2 (in macrophages) enhanced the IL-12/IL-10 expression ratio in the latter (Figure 7, A and B), which was further corroborated with enhanced cytotoxicity against tumor cells (Figure 7, C and D). Interestingly, concurrent disruption of both CSF1R and MIP2 expression from CAFs significantly enhanced these observations (Figure 7, A–D). Taken together, our findings unveiled a CAF-derived CSF1R-dependent mechanism of heterotypic macrophage M2 polarization. Our evidence suggests that CSF1R located in CAFs, once activated by CSF1 (secreted by tumor cells or tumor endothelium), leads to MIP2 secretion that subsequently signals through the CXCR2 receptor located on macrophages and enhances the IL-10/IL-12 ratio (which is functionally related with M2 polarization) (Figure 8).

## Discussion

TAMs are known to be a crucial component of several solid tumors, and their numbers are associated with poor prognosis and/or late stages of cancer (20). Their functions are mainly in favor of tumor progression, such as angiogenesis, T cell suppression, or even therapeutic resistance (21). It is not surprising, then, that they have long drawn attention as a potential target for cancer therapy. There are 3 recent strategies for TAM manipulation: a) elimination of macrophage tumor populations (such as clodronate, PLX7486), b) inhibition of their recruitment to tumors (for example, using carlumab or PF‑04136309), and c) reprogramming of TAMs toward the antitumor M1 phenotype. Total elimination of macrophages was previously shown to deprive tumors of favorable M1 populations, while inhibition of their infiltration was compromised by compensatory upregulation of alternative chemotactic mediators (22, 23). Specific targeting of protumor macrophages and reprogramming intratumor ones toward an antitumor phenotype seems to be the most promising approach. CSF1R inhibitors have been shown to repolarize TAMs toward an antitumor phenotype, enhance the efficacy of immunotherapies, and retard tumor progression, and they have a low-toxicity profile (20).

Stimulated by the great prevalence of macrophages in MPE, our study aimed to elucidate the importance of the CSF1/CSF1R axis in MPE formation. Our main findings are the following: a) CSF1R^+^ macrophages promote pleural fluid accumulation by enhancing vascular permeability, destabilizing tumor vessels, and favoring immune suppression. b) CSF1R inhibition limited MPE in vivo by reducing vascular permeability and neoangiogenesis and impeding tumor progression. c) CSF1Ri administration reduced fluid macrophage counts, attenuated macrophage-derived secretion of proangiogenic mediators, and reprogrammed TAMs toward an M1 phenotype. d) In addition, it reshaped the immune environment by reducing myeloid-suppressing populations, enhancing DC and CD8^+^ lymphocyte activation, limiting expansion of the suppressing CAFs, and reducing VEGF and MIP2 expression. e) Finally, CSF1R inhibition halted CAF-derived-MIP2 paracrine signaling in macrophages that would otherwise trigger the manifestation of suppressive and angiogenic properties.

To our knowledge, this is the first study to directly document the functions of CSF1R^+^ macrophages in MPE formation. As mentioned, TAMs are the most prevalent cell population in MPE (4–6), among which the M2 tumor-promoting subpopulation may be up to 70% (24). Pleural TAMs depend on CSF1/CSF1R activation for their M2 functions (12, 13), and high CSF1 pleural levels are associated with poor survival, implying an unfavorable role of CSF1/CSF1R signaling in the pleura (13). By generating macrophage-specific CSF1R-KO mice, we showed that CSF1R^+^ macrophages promoted MPE by enhancing hyperpermeability of vessels and destabilizing tumor endothelium (by reducing VE-cadherin). This is in accordance with previous in vitro data supporting a direct role of M2 macrophages on endothelial barrier integrity through VE-cadherin (25). Although vascular permeability in MPE has been extensively studied, most of the studies focus on intercellular interactions among endothelial cells. This study points out the importance of heterotypic endothelial-macrophage cell interactions in vessel integrity. Moreover, we demonstrated that CSF1R^+^ macrophages favored immune suppression mainly by limiting CD8^+^ T cell infiltration and differentiation, which is compatible with a previous study in CSF1-silenced colorectal tumors that presented increased CD8^+^ T cell attack (26). Taken together, our main observations suggest that CSF1R macrophages significantly contribute in MPE formation and could be therapeutically exploited to limit MPE.

We therefore proceeded with pharmacological targeting of CSF1R through a highly selective and clinically established inhibitor, BLZ945, currently under phase I/II clinical testing (ClinicalTrials.gov NCT02829723). CSF1Ri administration was very potent in limiting MPE and tumor progression. CSF1Ri significantly reduced vessel density and hyperpermeability. Fluid macrophages were massively reduced in both models, and the remaining ones were polarized toward an M1 antitumor phenotype (upregulation of Inos and downregulation of Arg1 and Mrc1). This observed depletion of macrophages by CSF1Ri is consistent with previous reports (27–30) and occurred in contrast with an absence of TAM reduction in models with genetic CSF1R depletion. Relative to this, although CSF1R is implicated in the survival and proliferation of macrophages (31), CSF1R blockade in vivo has not always eliminated them (32–34). The reason for these contradictory observations remains elusive. In fact, in our study, MCP-1, known to favor recruitment of monocytes (35), was reduced accordingly and could therefore (even partially) account for decreased macrophage recruitment. Nevertheless, the importance of this finding is that the reduction of pleural macrophage numbers is known to provide a survival benefit to patients with MPE (5, 7) and turns the effusion into a reservoir of antitumor responses that could limit tumor progression. In fact, our study and other recent work demonstrate that CSF1R inhibition in macrophages enhance DC and CD8^+^ T cell activation combined with a reduction of suppressive MDSC populations (13, 32, 36–40). Apart from the apparent benefit of relieving immune suppression, restoring T cell activity in the pleura has recently been clinically proven to improve survival and quality of life of patients with MPE (41). Moreover, CSF1Ri administration reduced several secreted mediators known to exert a pleiotropic role in MPE formation, such as VEGF and IL-6 (42).

Finally, we showed that CSF1R inhibition can limit stromal activation (as witnessed by FAPA reduction) and demonstrated a CSF1R-dependent mechanism of paracrine macrophage suppression. CAFs that are also known to bear the CSF1R (17) are now proven to be implicated in macrophage infiltration (43, 44) and suppression (19, 45). We demonstrated a CAF-macrophage heterotypic interaction mediated by CSF1R present on CAF’s surface. In specific, our evidence suggests that triggering of CSF1R in CAFs mediates MIP2 secretion that upon binding to its CXCR2 receptor on macrophages compromises their cytotoxicity (by upregulation of IL-10 and downregulation of IL-12) and favors their angiogenic actions (upregulating *Vegfa* and *Il6* expression). Although it is likely that more than one chemokine may be involved, we focused on MIP2 because a) it has been reported as a molecular crosstalk between these 2 cell types (45) and (b) we found that MIP2 expression by CAFs was affected by CSF1/CSF1R signaling. The proposed mechanistic model derived from our in vitro experiments is compatible with those derived from transgenic mice where genetic ablation of CSF1R in macrophages failed to attenuate tumor progression (Supplemental Figure 1C) and vessel density (Figure 1, D and F). This might therefore be associated with the remaining CSF1R signaling from CAFs that compromised the effectiveness of macrophages and upregulated their VEGF expression.

The uniformly fatal prognosis for patients with MPE creates a strong need for new therapeutic (rather than palliative) manipulations for pleural effusions. The study presented here underlines the importance of targeting the CSF1/CSF1R axis in limiting MPE formation, providing a rationale for further clinical evaluation.

In conclusion, the CSF1/CSF1R axis could serve as an appealing target for MPE treatment because a) it repolarizes and depletes M2 macrophages, which are known to be the prevailing populations in MPEs and associated with dismal prognosis (23); b) it mediates stromal cell–M2 macrophage interactions that favor tumor progression and angiogenesis; c) both cell targets (macrophages and CAFs) are less prone to drug resistance because of their genomic stability (23, 46); and d) several CSF1R inhibitors are currently available showing a satisfactory safety profile (20).

## Methods

### Cells lines and reagents

Murine LLCs and colon carcinoma MC38 cells were purchased from European Collection of Authenticated Cell Cultures (ECACC). Both cell lines used in the present study were maintained in DMEM (10% FBS) and were periodically monitored for mycoplasma presence by PCR. Bone marrow–derived macrophages were isolated from the marrow of C57BL/6 mice, as previously described (47). M1 phenotype activation was triggered by 24-hour incubation with LPS (2 μg/mL, MilliporeSigma). CAFs were isolated from LLC or MC38 tumors by FAPA antibody–loaded (CSB-PA008191, Cusabio Technology) magnetic beads (MojoSort Streptavidin Nanobeads, BioLegend) as previously reported (48).

Generation of MIP2-deprived CAFs and CXCR2-deprived macrophages was accomplished by transient transfection (Xfect polymer, Takara Bio) of the primary cells with 10 nM of Dicer-substrate short interfering RNAs (DsiRNAs) (mm.Ri.Cxcl2.13.3 and mm.Ri.Cxcr2.13.3 and negative control DsiRNA, Integrated DNA Technologies); 48 hours later, cells were used in the experiments described below.

BLZ945 was provided by Novartis Pharmaceuticals. BLZ945 was freshly prepared and formulated at a concentration of 12.5 mg/mL in vehicle consisting of hydroxypropyl-β-cyclodextrin (HPβCD, 20% w/v) dissolved in H_2_O and was given at a dose of 200 mg/kg by oral gavage once daily. This regimen has been previously shown to be well tolerated by mice and to cause no toxicity (32, 37). Murine CSF1 and IL-34 were purchased from BioLegend.

### In vivo studies

C57BL/6 mice were purchased from BSRC Alexander Fleming (Vari, Greece). The *B6.129P2-Lyz2^tm1(cre)Ifo^/J* (LysM-Cre, Cre expression is under the control of the endogenous *Lyz2* promoter; ref. 49) strain was provided by George Kollias (BSRC Alexander Fleming, Vari, Greece) upon permission of Irmgard Förster (University of Bonn, Bonn, Germany). The *B6.Cg-Csf1r^tm1.2Jwp^/J* (*CSF1R-floxed*) mice possessing *loxP* sites flanking exon 5 of the Csf1r gene were obtained from Melanie Greter (University of Zurich, Zurich, Switzerland). All mice were housed at the Animal Model Research Unit of Evangelismos Hospital (Athens, Greece) and received food and water ad libitum.

#### MPE mouse models.

For experimental MPE generation, 1.5 × 10^5^ LLCs or MC38 cells were intrapleurally injected into 8- to 10-week-old sex- and weight-matched syngeneic mice (50). Intrapleural delivery of cells was accomplished as previously described (51). Briefly, mice were anesthetized by a ketamine/xylazine mixture, and the area overlying the anterior and lateral chest wall was disinfected. A 1 cm wide skin incision was subsequently made on the left anterolateral thoracic area at the xiphoid level. Fascia and muscle were removed until the ribs were revealed and the pleural cavity became visible so that 50 μL of cell suspension (3 × 10^6^ cells/mL) could be easily injected through an intercostal space. The incision was closed using a 5-0 monofilament silk suture. Animals were given 1 mL (i.p.) of normal saline for hydration and monitored until they recovered. Animals were euthanized 13 days after injection; at this point, they were usually exhibiting respiratory distress and impaired motility. Lungs were harvested and visceral pleural foci were enumerated under a stereoscope (Stemi DV4). Pleural fluid was retrieved and quantified using gradual pipette aspiration. Total MPE nucleated cells were counted using a hemocytometer. Pleural vascular permeability was assessed using the in vivo Evans blue assay (52).

#### Generation of LysM-Cre^hemi^ CSF1R^fl/fl^ mice.

In order to create C57BL/6 mice whose macrophages were devoid of CSF1R, we crossed CSF1R-floxed mice with LysM-Cre strains. Littermates hemizygous for Cre and homozygous for *loxP* (*LysM-Cre^hemi^* *CSF1R^fl/fl^*) were consequently expected to have nearly complete deletion of the CSF1R gene in the myeloid lineage. Deletion in DCs was very low (16%) (49), and granulocytes did not express CSF1R, although mRNA was transcribed (53). The percentage of CSF1R-expressing cells among major immune populations present in the pleural fluid and tumors of *LysM-Cre^hemi^* *CSF1R^fl/fl^* and *LysM-Cre^–/–^* *CSF1R^fl/fl^* mice are presented in Supplemental Figure 8. Intermediate genotypes *LysM-Cre^hemi^* *CSF1R^wt/wt^*, *LysM-Cre^–/–^* *CSF1R^fl/fl^*, and *LysM-Cre^hemi^* *CSF1R^wt/fl^* were also tested in preliminary studies to ascertain that Cre-recombinase did not contribute to or interfere with the phenotype of interest. Thereafter, *LysM-Cre^–/–^* *CSF1R^fl/fl^* mice were used as controls. Mice bearing the desired phenotypes were selected upon genotyping by PCR (described below).

#### MPE therapeutic study.

MPE-bearing mice were administered (by oral gavage) the CSF1R inhibitor BLZ945 at the dose of 200 mg/kg once daily, according to a previous study (28). As a control, mice treated with vehicle alone were used. Treatment was started the fourth day after tumor cell injection since pleural tumors are already evident at this time point (ref. 54, Supplemental Figure 9, A and B).

### Genotyping of LysM-Cre and CSF1R-floxed mice

DNA extraction from mouse tails was performed using the NucleoSpin tissue mini kit (Macherey-Nagel) according to the manufacturer’s instructions. LysM-Cre mice were genotyped by PCR using the primer set forward, 5′ CTTGGGCTGCCAGAATTTCTC 3′; reverse, 5′ CAGGTATGCTCAGAAAACGCCT 3′. The PCR condition was 94°C for 3 minutes; 29 cycles of 93°C for 45 seconds, 56°C for 45 seconds, 72°C for 45 seconds; and final extension at 72°C for 10 minutes. Primers for genotyping CSF1R-*floxed* mice were intron 5, 5′ GCCACCATGTGTCCGTGCTT 3′; exon 5, 5′ ACCCAGAGCCCCCACAGATA 3′. The PCR condition was 95°C for 8 minutes; 35 cycles of 95°C for 45 seconds, 58°C for 45 seconds, 72°C for 1 minute; and final extension at 72°C for 5 minutes.

### Flow cytometry

Tumor and pleural immune cells were fixed, permeabilized, and stained with anti-CD45 (clone 30-F11), anti-CD11b (clone M1/70), anti-F4/80 (clone BM/8), anti-CD206 (clone C068C2), anti-Ly6C (clone HK1.4), anti-Ly6G (clone RB6-8C5), anti-CD11c (clone HL3), anti-MHCII (clone M5/114.15.2), anti–IL-10 (clone JES5-16E3), anti–IL-12 (clone C15.6), anti-CSF1R (clone AFS98), anti-CD3 (clone 145-2C11), anti-CD4 (clone GK1.5), anti-CD8 (clone YTS1567.7), anti-Foxp3 (clone MF14), and anti–granzyme B (clone QA18A28) (all purchased from BioLegend). Inflammatory cells were selected based on their forward- and side-scatter profiles and their CD45^+^ staining. Specifically, macrophage populations were characterized as CD11b^+^Ly6G^–^Ly6C^lo^ F4/80^+^, Mo-MDSCs as CD11b^+^Ly6G^–^Ly6C, polymorphonuclear MDSCs as CD11b^+^Ly6G^+^Ly6C^lo^, and activated DCs as CD11c^+^MHCII^+^ cells. M2 macrophage phenotype was determined as F4/80^+^CD206^+^. In addition, M1/M2 phenotypes were evaluated according to the macrophage IL-12/IL-10 expression ratio. Total numbers of CD3^+^CD4^+^ and CD3^+^CD8^+^ lymphocytes were also enumerated. Tregs were determined as CD4^+^Foxp3^+^, as well as activated CD8^+^ T cells by granzyme B expression. The flow cytometry data were acquired using BD FACSCanto II flow cytometer and analyzed by FlowJo software.

### Immunohistochemistry and immunofluorescence analyses

FFPE tumor tissues were stained using primary antibody rabbit anti-proliferating cell nuclear antigen (PCNA; clone D3H8P, Cell Signaling Technology) at 1:8000 dilution for evaluation of tumor cell proliferation. Biotinylated goat anti-rabbit IgG (PK-610), ABC complex kit, and DAB substrate kit (Vector Laboratories) were used for detection and visualization. Tumor cell apoptosis was estimated by TUNEL assay, as previously described (55).

Evaluation of CAFs was performed upon FAPA staining of tumor tissue paraffin sections using an anti-FAPA antibody (1:200) (CSB-PA008191). FAPA-positive brown area was quantified by Fiji (ImageJ, NIH) software and the color segmentation plugin (Daniel Sage, 2008, http://bigwww.epfl.ch/sage/soft/colorsegmentation/) using the K-means algorithm clustering method.

For immunofluorescence analysis, tumor tissues were fixed in 4% paraformaldehyde overnight at 4°C and then transferred to 30% sucrose at 4°C. Cryosections were stained for the presence of CD31 (clone MEC 13.3, BD Biosciences) and VE-cadherin (clone ab33168, Abcam) (for endothelial and intercellular junctions staining, respectively) and were mounted using Fluoroshield Mounting Medium with DAPI (ab104139, Abcam). Fluorescence was quantified using Fiji (ImageJ, NIH) software.

### Macrophage gene expression analysis

#### In vivo angiogenic and functional macrophage gene expression.

Macrophages released from tumor tissues upon collagenase (1 mg/mL) digestion and pleural fluid macrophages were isolated by magnetic streptavidin beads bearing anti-F4/80 antibody (clone BM8, MojoSort beads, BioLegend). TAMs were characterized according to mRNA expression levels of key M1-associated (*Inos*, *Fizz*, *Tnfa*) and M2-associated (*Mrc1*, *Arg1*) genes using real-time PCR. Angiogenic potential of TAMs was determined upon measurement of *Angpt2*, *Il6*, and *Vegfa* mRNA levels. RNA was extracted using the NucleoSpin RNAplus kit (Macherey-Nagel). For cDNA synthesis, the PrimeScript first strand cDNA synthesis kit (Takara, Clontech) was used with 1 μg of RNA. Assays were performed in duplicate and relative expression was normalized to *Gapdh* for each sample.

Isolated CAFs from MC38 or LLC tumors (treated with vehicle or 6700 nM CSF1Ri overnight) or shMIP2 CAFs were seeded at 2 × 10^5^ cells/well in 24-well plates and subsequently cocultured with macrophage vector or macrophage shCXCR2 for 48 hours. Macrophages were subsequently purified from the coculture using magnetic beads, and mRNA levels of central angiogenic genes (*Vegfa*, *Il6*, and *Angpt2*) were quantified by real-time PCR as aforementioned.

### Cytokine and chemokine quantification

Pleural fluid and tumor tissue lysates were analyzed for the presence of IL-6, TNFA, and MCP-1 (PeproTech) and VEGF, OPN, and MIP2 (R&D Systems) by ELISA according to the manufacturer’s instructions. Tumor tissue cytokine levels were normalized to total tissue protein.

Isolated CAFs from LLC or MC38 tumors were seeded at 2 × 10^5^ cells/well in 24-well plates and treated with vehicle, 6700 nM CSF1Ri, 20 μg/mL CSF1, or 20 μg/mL IL-34 or pretreated with CSF1Ri for 2 hours and then CSF1; 24 hours later, supernatants were collected and MIP2 levels were quantified by ELISA (R&D Systems). Results were normalized to total protein.

### In vitro cell viability

For assessing cell viability, murine LLC and MC38 adenocarcinoma cells were seeded at 3 × 10^3^ cells/well in 96-well plates. Media were removed 24 hours later and replaced with fresh complete medium containing vehicle or escalating doses of BLZ945 (6.7–6700 nM). Cell viability was subsequently measured by XTT reduction at 450 nm (XTT cell viability assay kit, Biotium Inc.).

### CAF, macrophage, and tumor cell cocultures

Activated CAFs were isolated from LLC or MC38 tumors as aforementioned, seeded at 2 × 10^5^ cells/well in 24-well plates, and treated with vehicle or 6700 nM CSF1Ri overnight. Media were then aspirated and CAFs were subsequently loaded with 7 × 10^4^ tumor cells/well; 8 hours later 2 × 10^5^ M1 macrophages/well (prepared by overnight incubation with 2 μg/mL LPS) were added to the tumor cell–CAF cocultures. CD45^+^CD11b^+^F4/80^+^ macrophages were analyzed for IL-10 and IL-12 expression using flow cytometry 24 hours later. To assess macrophage cytotoxicity, experiments were repeated with tumor cells that were preloaded with 4 μM BCECF-AM (MilliporeSigma) for 30 minutes at 37°C. Tumor cell counts were subsequently assessed upon cell lysis by 485/525 nm measurement.

### Statistics

All values are presented as mean ± SEM. Differences between groups were evaluated using the 2-tailed Student’s *t* test or 1-way ANOVA with Bonferroni’s post hoc test for multiple comparisons, as appropriate. *P* values less than 0.05 were considered significant. Statistical analysis was performed using SPSS v.13.0.0 (IBM).

### Study approval

Experiments were approved by the Veterinary Administration Bureau, Prefecture of Athens, Greece (decision 7726/11.2016), in compliance with the national law and the EU Directives.

## Author contributions

CNK, SFM, PCV, AGP, and MPI performed experiments; SFM and ITK were responsible for the study conception and design; SFM, PCV, AGP, MPI, CNK, KKP, and ITK performed analysis and interpretation; SFM and ITK drafted the manuscript, providing important intellectual content.

## Supplementary Material

Supplemental data

## Version 1 03/22/2022

Electronic publication
