# Supplementary material for: CSF1/CSF1R signaling mediates malignant pleural effusion formation
Source: JCI Insight. 2022 Mar 22;7(6):e155300. doi: 10.1172/jci.insight.155300 (PMC8986064; doi:10.1172/jci.insight.155300)

Supplementary Data

Supplementary Figure 1. CSF1R macrophages were successfully depleted in both pleura and tumors *of* $LysMCre^{hemi}CSF1R^{fl/fl}$ mice. CSF1R+ macrophage ablation did not affect tumor foci numbers. Mice whose macrophages are devoid of CSF1R ($LysMCre^{hemi}CSF1R^{fl/fl}$) or not ($LysMCre^{-/-}CSF1R^{fl/fl}$) were intrapleurally injected with $1.5x10^5$ LLC or MC38 murine adenocarcinoma cells. Animals were euthanized 13 days later. CSF1R+ macrophages (CD11b+/CD45+/F4/80+) were quantified among pleural (A) and tumor cells (B) using flow cytometry. LLC: $LysMCre^{hemi}CSF1R^{fl/fl}$ n=6, $LysMCre^{-/-}CSF1R^{fl/fl}$ n=5. MC38: $LysMCre^{hemi}CSF1R^{fl/fl}$ n=5, $LysMCre^{-/-}CSF1R^{fl/fl}$ n=5. *p<0.05 compared to $LysMCre^{hemi}CSF1R^{fl/fl}$ by 2-tailed students' T-test. (C) Lungs were harvested and visceral pleural foci were counted under a stereoscope. Data presented as mean±SEM, LLC $LysMCre^{hemi}CSF1R^{fl/fl}$ n=15, $LysMCre^{-/-}CSF1R^{fl/fl}$ n=10. MC38: $LysMCre^{hemi}CSF1R^{fl/fl}$ n=10, $LysMCre^{-/-}CSF1R^{fl/fl}$ n=7. *p<0.05 compared to $LysMCre^{hemi}CSF1R^{fl/fl}$ by 2-tailed students' T-test.

Supplementary Figure 2. % CSF1R-expressing cells **in main immune populations of tumors and pleural fluid of $LysMCre^{hemi}CSF1R^{fl/l}$ and $LysMCre^{-/-}CSF1R^{fl/fl}$ mice bearing LLC and MC38-induced MPE.** Pleural and tumor (A) CD45+CD11b+F4/80+ macrophages, (B) CD45+CD11c+ DCs, (C) CD45+CD11b+ LY6C+ monocytes, (D) CD45+CD11b+ LY6G+granulocytes and (E) CD45+CD3+ lymphocytes of LLC (left) and MC38 right) induced MPE bearing mice were quantified in pleural fluid and tumors of $LysMCre^{hemi}CSF1R^{fl/l}$ and $LysMCre^{-/-}CSF1R^{fl/fl}$ mice. LLC $LysMCre^{hemi}CSF1R^{fl/fl}$ n=5-8, $LysMCre^{-/-}CSF1R^{fl/fl}$ n=5-8 MC38: $LysMCre^{hemi}CSF1R^{fl/fl}$ n=5-8, $LysMCre^{-/-}CSF1R^{fl/fl}$ n=5-8. *p<0.05 compared to $LysMCre^{hemi}CSF1R^{fl/fl}$ by 2-tailed students' T-test.

Supplementary Figure 3. Selective macrophages knockdown of CSF1R gene did not affect macrophage recruitment in the pleural space or tumors, but inhibited their polarization towards an M2 phenotype. Pleural (A) and tumor (B) macrophages were quantified upon CD45, CD11b, F4/80 staining using flow cytometry. IL12/IL10 expression ratio (indicative of M1/M2 polarization) was also determined among pleural (C) and tumoral (d) macrophages. LLC $LysMCre^{hemi}CSF1R^{fl/fl}$ n=10, $LysMCre^{-/-}CSF1R^{fl/fl}$ n=6 MC38: $LysMCre^{hemi}CSF1R^{fl/fl}$ n=5 $LysMCre^{-/-}CSF1R^{fl/fl}$ n=5. *p<0.05 compared to $LysMCre^{hemi}CSF1R^{fl/fl}$ by 2-tailed students' T-test.

Supplementary Figure 4. CSF1R+ macrophages promote immune suppression by enhancing MDSCs populations and reducing DC and CD8 T cell activation. Pleural (left) and tumor (right) abundance of Myeloid Derived Suppressor Cells (MDSCs) of the monocytic (CD11b+/Ly6C+) (A) and granulocytic (CD11b+/Ly6G+) (B lineage was determined in $LysMCre^{hemi}CSF1R^{fl/fl}$ or $LysMCre^{-/-}CSF1R^{fl/fl}$ animals by flow cytometry. (C) Total and (D) Activated (MHCII+) dendritic cells (CD45+/CD11C+) (E) total CD8 T cell numbers and (F) activated (granzyme-B+) CD8+ lymphocytes were also evaluated in pleural fluid and tumors of animals with CSF1R+ and CSF1R- macrophages. Data presented as mean±SEM, n=5-6, for all groups *p<0.05 compared to $LysMCre^{hemi}CSF1R^{fl/fl}$ by 2-tailed students' T-test.

Supplementary Figure 5. CSF1R+ macrophage ablation does not significantly alter the pleural and tumor cytokine mileu. Pro-inflammatory mediators central to MPE formation (IL-6, VEGF, MCP-1, TNF-alpha, OPN and MIP-2) were determined in pleural fluid (a, b) and tumor lysates (c, d) of LLC or MC38 tumor bearing CSF1R+macros or CSF1R-macros-mice. Data presented as mean±SEM, n=5-6, for all groups *p<0.05 compared to *LysMCre^{hemi}CSF1R$^{fl/fl}$* by 2-tailed students' T-test.

Supplementary Figure 6. Pharmacological targeting of CSF1R attenuates tumor foci formation. Representative pictures (taken under a stereoscope equipped with a digital camera) of lungs retrieved from CSF1Ri or vehicle treated mice. Magnification: 48x. Arrows depict tumor foci.

Supplementary Figure 7. CSF1R blockade does not affect the viability of LLC or MC38 tumor cells. (a,b) LLC or MC38 murine adenocarcinoma cells were seeded at 3x10^3 cells/well in 96-well plates. The day after, cells were treated with vehicle or CSF1Ri (6.7-6700 nM) and 24 h later cell viability was measured by XTT reduction. Data are presented as mean±SEM, n=6 for each group, *p<0.05 compared to vehicle by 2-tailed students' T test.

Supplementary Figure 8. CSF1R+ macrophage ablation does not affect CAF populations. CAFs in LLC or MC38 tumors from *LysMCre^{hemi}CSF1R$^{fl/fl}$ and LysMCre$^{-/-}$CSF1R$^{fl/fl}$* mice were visualized upon FAPA staining using immunohistochemistry. (A) Representative pictures and (B) results of image analysis. Data presented as mean±SEM, n=5 for all groups *p<0.05 compared to *LysMCre^{hemi}CSF1R$^{fl/fl}$* by 2-tailed students' T-test.

Supplementary Figure 9. Tumor dissemination on the 4th day upon intrapleural tumor cell implantation. (A, B) Representative pictures (taken under a stereoscope equipped with a digital camera) of tumor foci present on the lungs (A, B) of LLC injected mice. Magnification (A, B): 48X. Arrows depict tumor foci.

Supplementary Figure 1

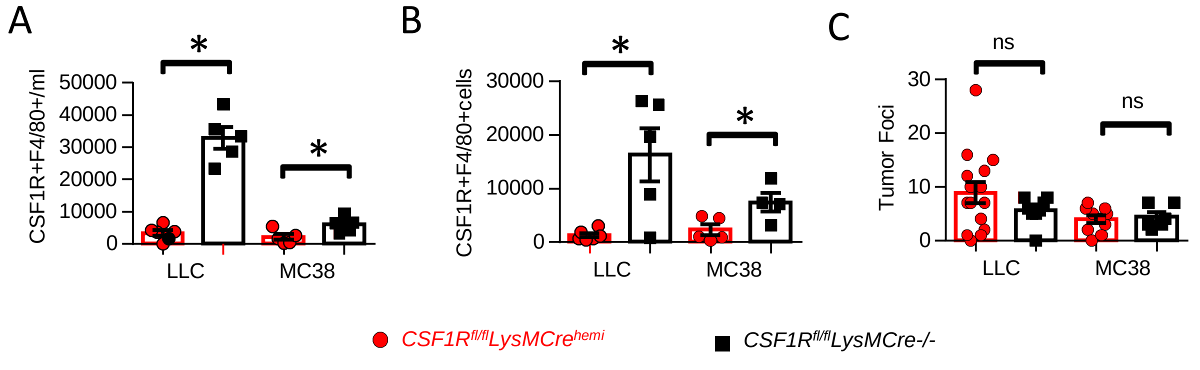

Supplementary Figure 2

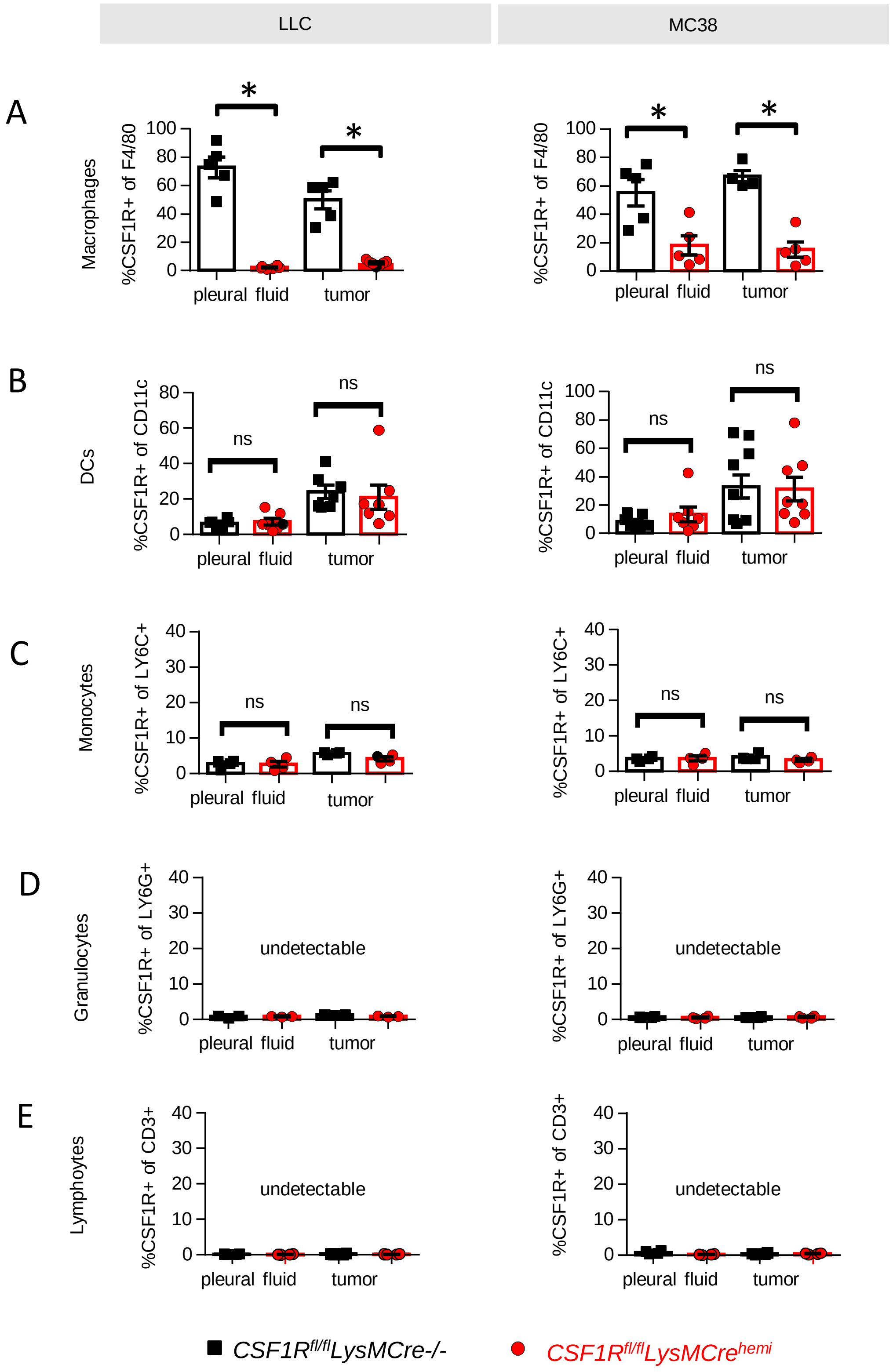

Supplementary Figure 3

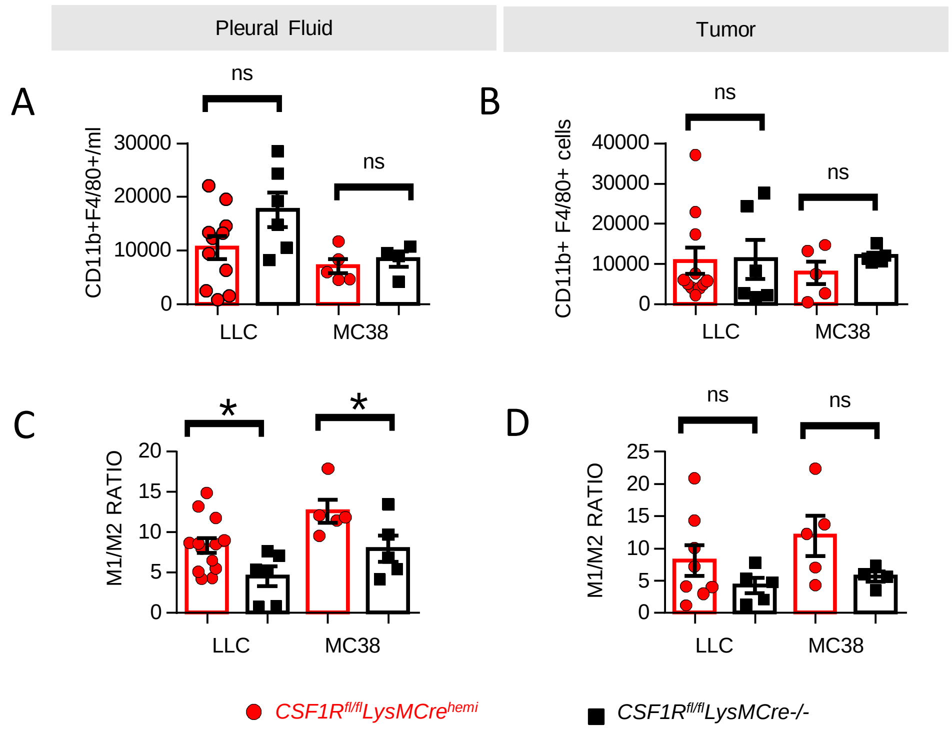

Supplementary Figure 4

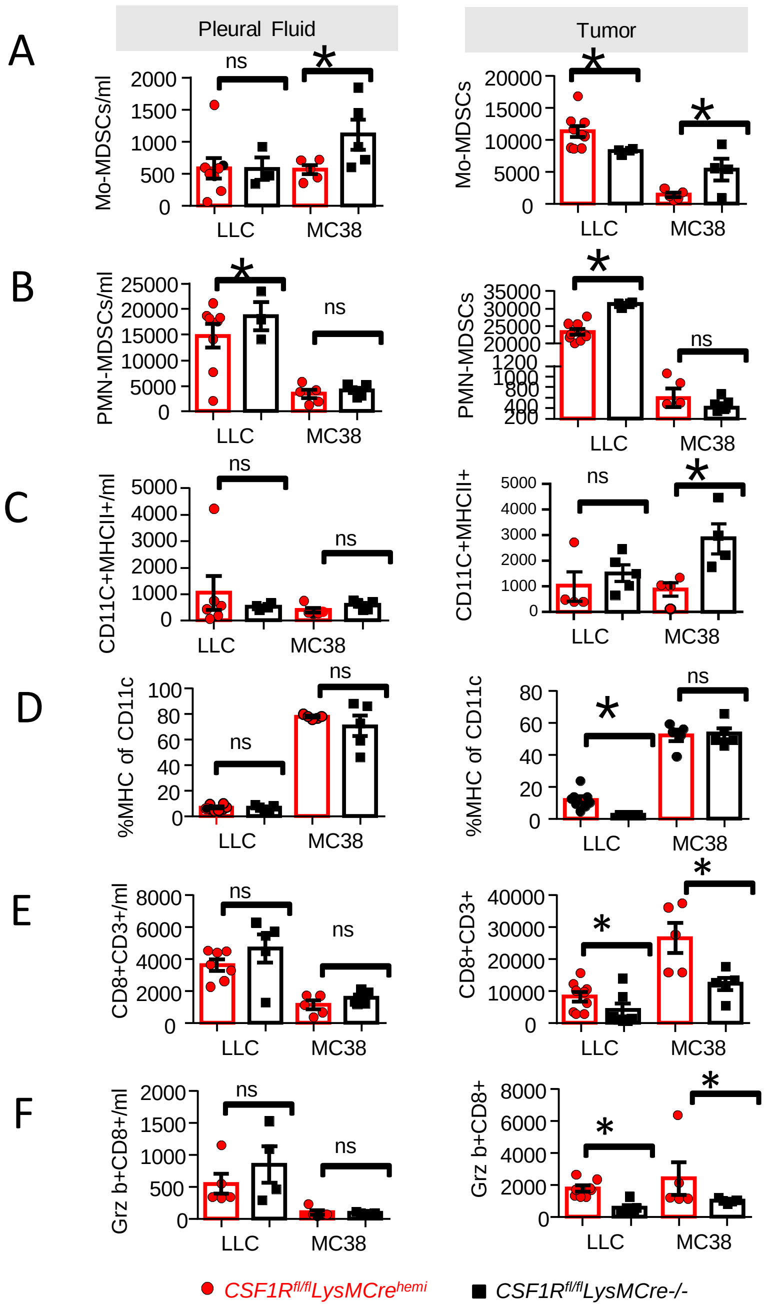

Supplementary Figure 5

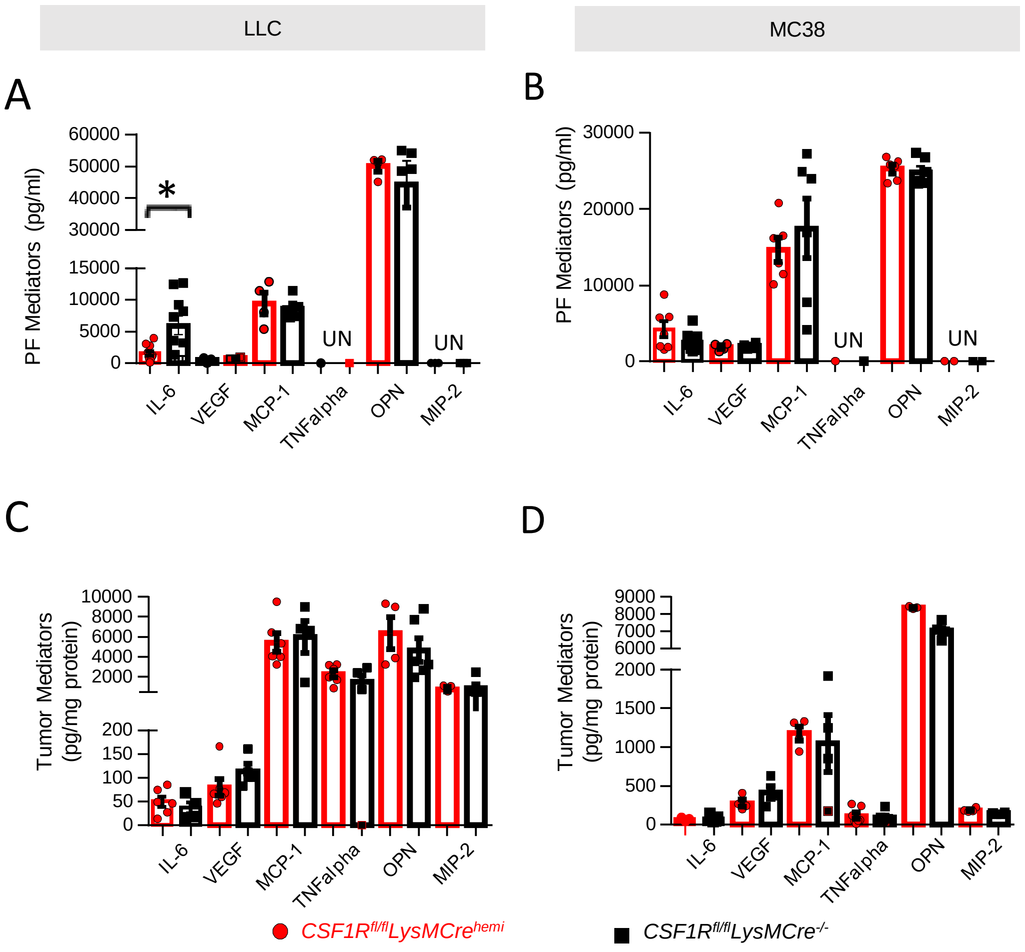

Supplementary Figure 6

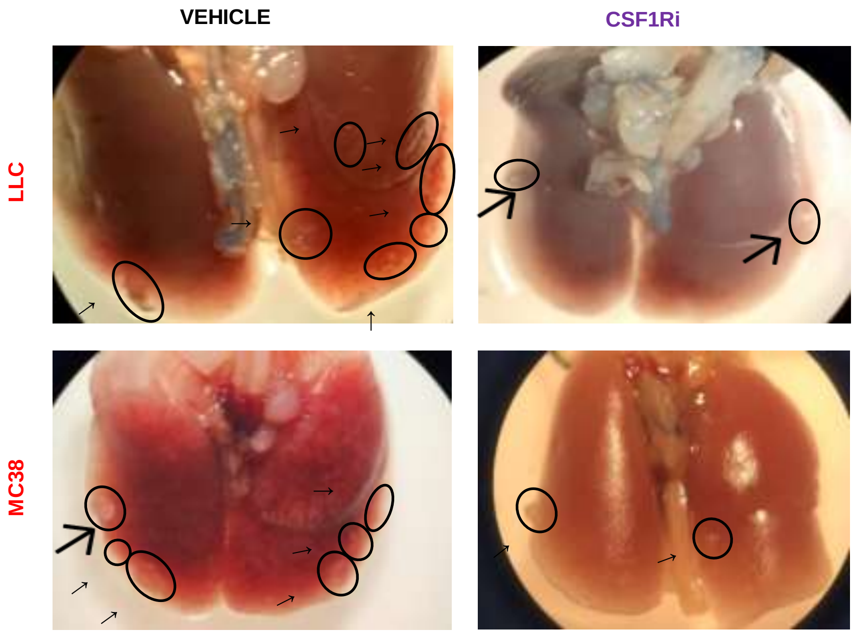

Supplementary Figure 7

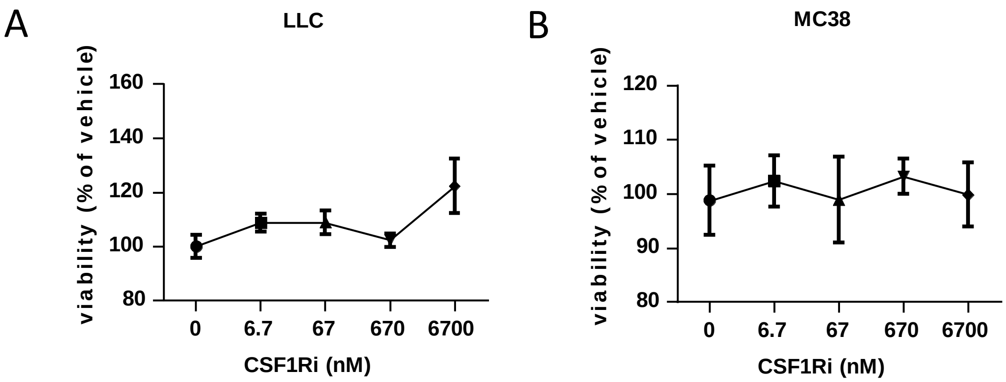

Supplementary Figure 8

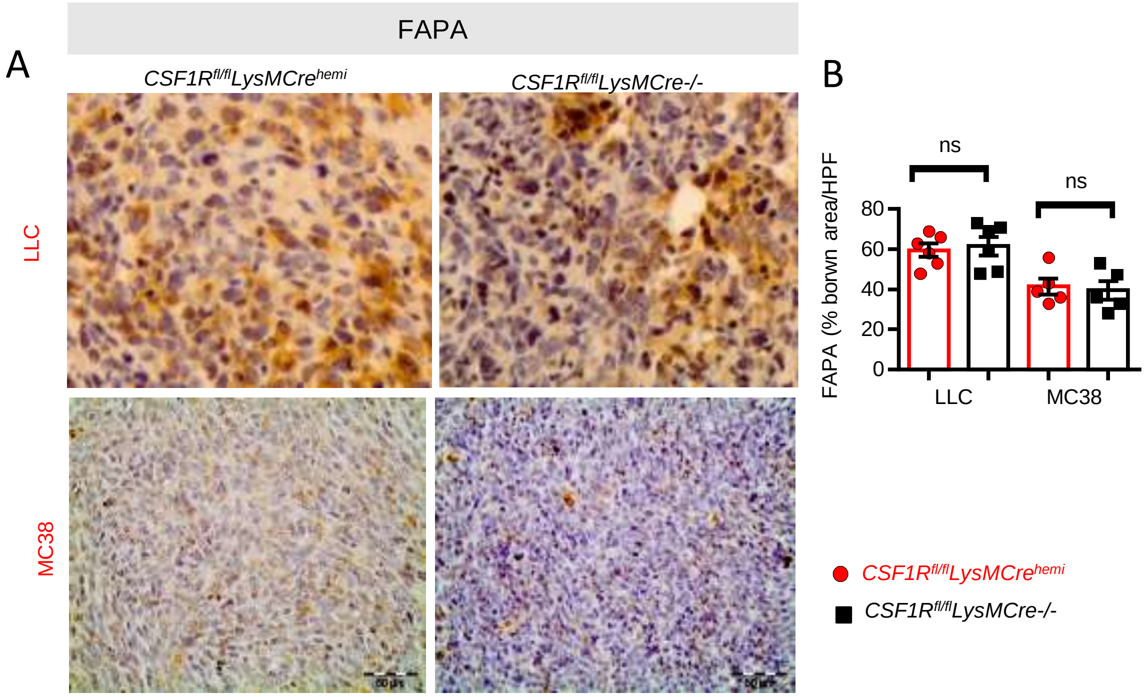

Supplementary Figure 9

A B

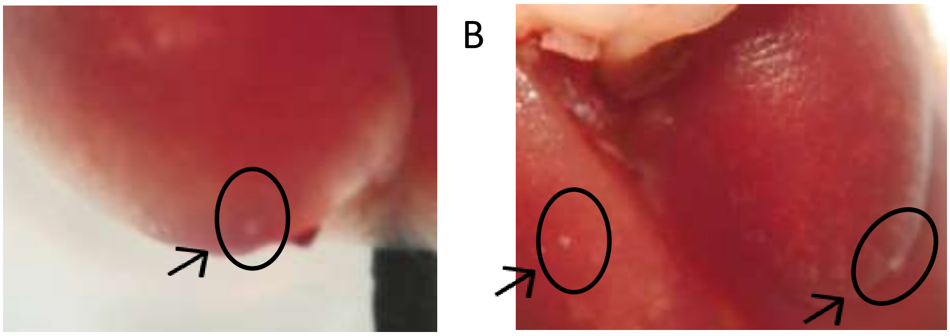

Supplement: Supplemental data [file jciinsight-7-155300-s147.pdf]
